# Effect of the Rolling Process on the Properties of the Mg/Al Bimetallic Bars Obtained by the Explosive Welding Method

**DOI:** 10.3390/ma16216971

**Published:** 2023-10-30

**Authors:** Sebastian Mróz, Karina Jagielska-Wiaderek, Andrzej Stefanik, Piotr Szota, Marcin Wachowski, Robert Kosturek, Marta Lipińska

**Affiliations:** 1 Faculty of Production Engineering and Materials Technology, Czestochowa University of Technology, 42-201 Czestochowa, Poland; k.jagielska-wiaderek@pcz.pl (K.J.-W.); andrzej.stefanik@pcz.pl (A.S.); piotr.szota@pcz.pl (P.S.); 2 Faculty of Mechanical Engineering, Military University of Technology, 2 Gen. S. Kaliskiego St., 00-908 Warsaw, Poland; marcin.wachowski@wat.edu.pl (M.W.); robert.kosturek@wat.edu.pl (R.K.); marta.orlowska@wat.edu.pl (M.L.)

**Keywords:** explosive welding, rolling, bimetallic bars, corrosion properties, microstructures

## Abstract

This study aims to analyze the influence of the rolling process on the microstructure and corrosion properties of the Mg/Al bimetallic bars obtained by the explosive welding method. The bars investigated were rolled using two different types of rolling: classical rolling (Variant I) and modified rolling (Variant II). Two different temperatures (300 °C and 400 °C) for each of the variables were applied as well. In this study, rods with an aluminum plating layer constituting 16.8% of the cross-sectional area and an average thickness of about 0.93 mm were investigated. Based on the revealed results, it was found that after the rolling process, the material shows clearly lower values of both i*_cor_* and current in the passive range. In the joint zone of Mg/Al rods rolled at 400 °C, Al_3_Mg_2_ and Mg_17_Al_12_ intermetallic phases are distinguished, localized next to the Mg core, and characterized by columnar, coarser grains. In the transition zone closer to the Al layer, only the Al_3_Mg_2_ phase is revealed, characterized by a refined, small grain size.

## 1. Introduction

Thanks to their high specific strength, magnesium alloys are one of the most promising structural materials for durable, lightweight constructions, both on land and in vehicles. The limited use of magnesium alloys in products has poor corrosion resistance [1,2]. These are also products made of protective alloys that are covered with a coating that increases the efficiency of the device [3,4]. Thus, products made of magnesium alloys are protected against corrosion by using aluminum layers or coatings [5,6]. Aluminum layers are a promising solution that increases the corrosion resistance of the magnesium alloy without causing a significant increase in the weight of the finished products. Aluminum layers, in addition to having very good resistance to corrosion in inert media, are resistant to mechanical damage and abrasive wear. The main methods that increase the corrosion resistance of magnesium alloys are laser surface alloying [7,8] and thermo-chemical treatment [9]. These methods are commonly used to produce Al-enriched layers. The disadvantage of these methods is obtaining very thin coatings with a maximum thickness of ~10 µm. Such thin layers are easy to mechanically damage and deteriorate corrosion resistance [8,9]. Therefore, it is necessary to use methods enabling the production of Al layers of greater thickness. Such methods include diffusion bonding [10,11], explosion welding [12,13], hot pressing [14], extrusion [15,16], flat rolling [17,18] or groove rolling [19,20], forging [21,22], and twin roll casting [23]. Some of the ones mentioned do not guarantee the appropriate quality of the connection or, in the case of bimetal rods, even the distribution of the plating layer around the circumference [17,24]. Bimetal rods can be produced by extrusion methods [15,16,25] or rolling in passes [24,26]. The feedstock for these processes is produced directly in the processes themselves, as in the case of extrusion [15,16] or using the casting method [19] or explosive welding [26].

The plating process can be achieved through explosive welding, which allows for the elimination of the problem of intermetallic phase formation between the aluminum layer and the magnesium core. In the previous article, the authors demonstrated that the explosive welding process allows for the production of magnesium rods plated with aluminum, which can then be further plastically formed [27,28]. In the mentioned study, a 30% aluminum layer content in the bimetal was considered, which has been reduced to 15% in subsequent development work.

In order to achieve a uniform distribution of the plating layer on the circumference and length of the rod, a combination of explosive welding and subsequent rolling in elongating dies is employed [19,29,30]. This combination of methods has been verified in studies involving explosive welding followed by the rolling of circular bimetallic rods, steel/Cu [29], and Al/Cu [30]. In recent years, this combination of methods has also been applied to obtain Mg/Al round bimetallic rods [19,20,27]. It is particularly essential to design the shapes of the grooves that, on the one hand, ensure joint quality and, on the other hand, provide an even distribution of the plating layer around the circumference and along the length of the rod. Rolling the rods in the passes occurs in a spatially deformed state, causing uneven plastic flow of individual layers, resulting in thinning of the plating layer and, in extreme cases, layer delamination [30].

In a previous work by the authors [27], the influence of process parameters (mainly temperature) on the distribution of the plating layer on the circumference of the rods was determined. In the cited work, the impact of groove shapes and temperature on the distribution of the plating layer thickness, ovality of the rods, and joint quality were studied. The cited work also examined the corrosion resistance of Mg/Al bimetallic rods. It was shown that the use of modified passes and a reduced rolling temperature of 300 °C provided a more even distribution of the plating layer, increased joint strength, and greater corrosion resistance compared to rods rolled with classical circular-oval-circular passes. In this study, only one part of the plating layer in the cross-section of the rod, which was approximately 28%, was investigated. The applied plating layer was relatively large, and even with a less favorable distribution of the plating layer, it ensured high corrosion resistance of the magnesium core.

In this work, bimetallic Mg/Al rods were used for this study, with the aluminum plating layer constituting approximately 15% (16.8) of the cross-sectional area of the rod. The rolling process was conducted at two temperatures, 300 °C and 400 °C, using two circular-oval-circular pass arrangements: classical (Variant I) and modified (Variant II). The billet was produced using the explosive welding method.

## 2. Material and Research Methodology

Ten sets of samples consisting of aluminum tubes (grade AA1050) and magnesium alloy rods (grade AZ31) were prepared for testing. The chemical composition of the materials used for testing is shown in Table 1.

The initial dimensions of pipes and bars used for explosive welding are listed in Table 2.

**Figure 1:**
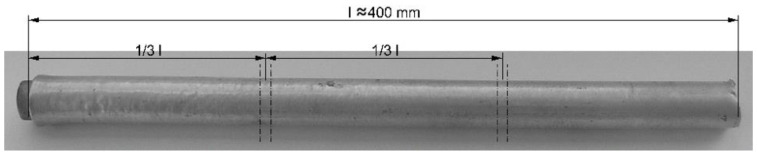
The shape and dimensions of the Mg-Al bimetallic bar after explosion welding.

For the explosive welding of Mg-Al rods, the ammonal explosive was used, which consisted of a mixture of ammonium nitrate and fuel oil. The casing of the prepared explosive welding system was a paper tube with an internal diameter of 50 mm and a wall thickness of 5 mm. The aluminum pipe was covered with a layer of approx. 0.5 mm-thick polyethylene. The thickness of the explosive layer was about 12 mm. After detonation, straight bimetallic rods were obtained without distortions or narrowings. As a result of explosive welding in the bars, the aluminum layer was slightly thickened, while the length of the aluminum pipe was slightly shortened. The shape of an exemplary Mg-Al bimetallic rod after explosive welding is shown in Figure 1. The figure also shows the places of sampling for further joint strength and microstructural tests.

**Figure 2:**
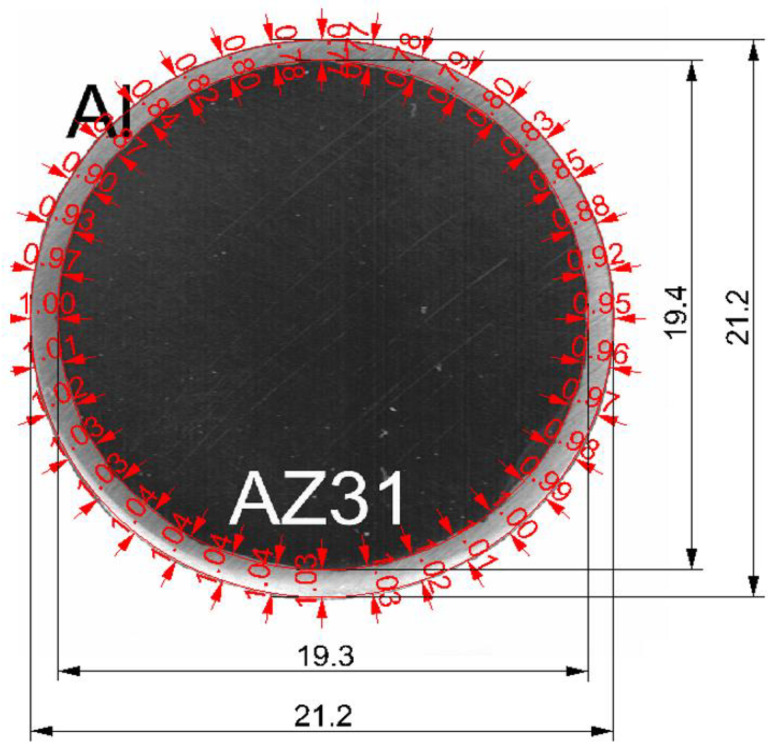
Shape and dimensions of Mg-Al bimetallic samples after explosive welding (cross-section)—light microscope image.

Examples of cross-sections and dimensions of Mg-Al bimetallic bars after explosive welding are shown in Figure 2 and presented in Table 3.

The data presented in Figure 2 and Table 3 show that after explosive welding, slight differences were obtained in the thickness of the aluminum layer on the circumference of the magnesium core. The average thickness of the aluminum layer was 0.93 mm, and the share of the aluminum layer in the cross-section of the bimetallic rod was about 16.8%. The obtained values of the non-uniformity of the plating layer were characterized by the parameter Kplat, which is the ratio of the maximum thickness to the minimum thickness of the Al layer. The non-uniformity of the plating layer distribution around the perimeter of the core for Mg/Al bars was Kplat = 1.35.

### 2.1. Rolling of Mg-Al Bimetallic Rods

A duo rolling mill with a nominal working roll diameter of 150 mm and a roll barrel length of 170 mm was used to carry out the experimental tests of the bimetallic bar rolling process. Each roller was driven individually by an AX monitor with a nominal power of 7.5 kW through a reducer with a ratio of 1:22.4 and a drive shaft. The charge was heated in the LAC KC 120/14 resistance chamber furnace. Prior to rolling in the individual pass, the strand was reheated and bent at 90° before being fed into the pattern. The rolling speed was 0.2 m/s. Grooves of cross-sections were taken after each culvert. During rolling, the value of the total pressure force of the metal on the rolls was recorded using strain gauges.

Two systems of elongating grooves were used for rolling:

1. Variant I: classic pattern of elongating cuts, circle-oval-circle.
2. Variant II: modified system of multi-radium elongating circle-oval-circle patterns.

**Figure 3:**
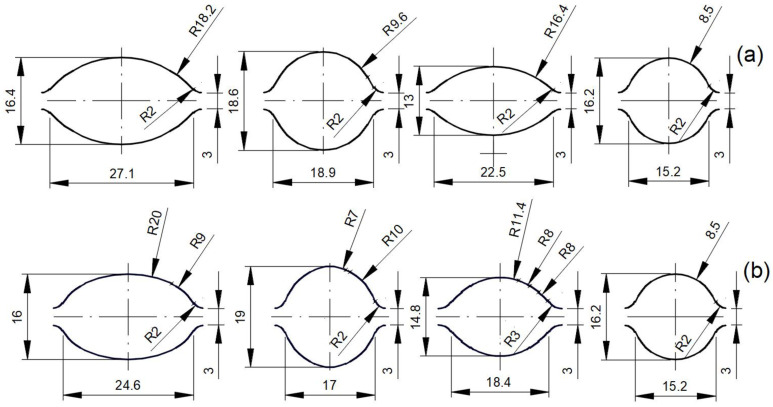
The shape and dimensions of the designed elongating passes are: (**a**) classic system (variant I); (**b**) multi-radial modified system (variant II) [27].

The shape of the grooves used for the rolling process is shown in Figure 3. Types and designations of tested materials are presented in Table 4.

**Figure 4:**
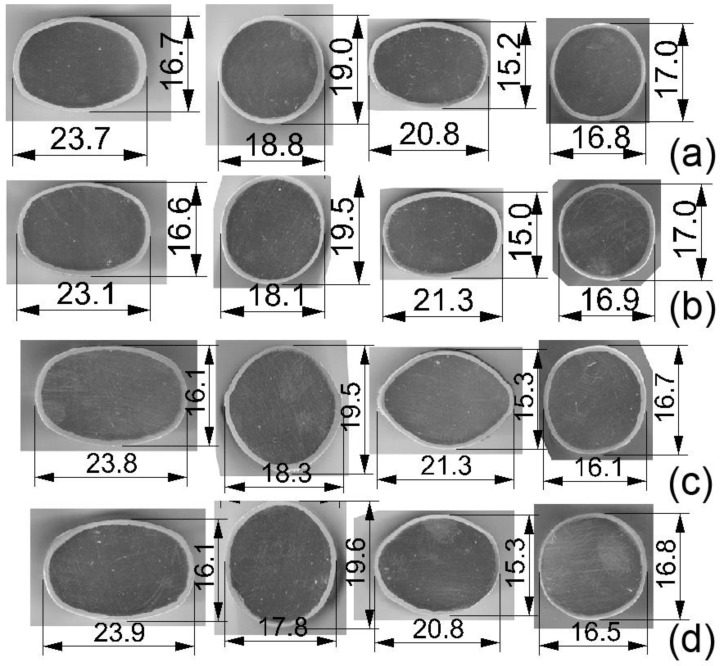
The shape of the finished Mg-Al bimetallic rods after each penetration: (**a**) Rolling in a classic arrangement of elongating passes at 300 °C (Series I), (**b**) classic arrangement of elongating passes at 400 °C (Series II), (**c**) modified arrangement of elongating passes at 300 °C (Series III), (**d**) modified arrangement of elongating passes at 400 °C (Series IV)—light microscope image.

For both patterns, rolling was carried out in 4 passes, obtaining round bars with a diameter of approx. 17 mm. The shape of the finished bars is shown in Figure 4. The bimetallic rods were rolled at two temperatures: 300 and 400 °C.

Based on the data presented in Figure 4 and Table 5, it is evident that the newly developed pass arrangement has a favourable impact on the distribution of the Al layer thickness. An improvement in the Al layer thickness distribution of bimetallic rods has been achieved, with a change of approximately 5% observed in the analysed cases. Greater difficulties in achieving a uniform plating layer occur when rolling Mg/Al bimetallic rods at a temperature of 400 °C. This is due to the yield stress ratio of Mg compared to Al. At a temperature of 400 °C, the ‘flow’ effect of the coating from the core is more pronounced. Therefore, the rolling of bimetallic rods should be conducted at a temperature where the yield stresses of the components are most closely matched; in this case, it is 300 °C.

### 2.2. Microstructural Studies

Microstructural studies of bimetallic rods included observations using a light microscope (Olympus LEXT OLS 4100) and a scanning microscope (JEOL JSM-6610). The samples were embedded in the resin, sanded with 80, 320, 500, 800, 1200, 2400, and 4000 grit papers, and then polished with 3 µm and 1 µm diamond paste. In order to reveal the microstructure of the AZ31 alloy, the specimens were etched with a reagent composed of 95 mL of ethanol, 10 mL of acetic acid, and 5 g of picric acid (etching time: 15 s). Revealing the microstructure of AA1050 was realized using 1% HF with an etching time of 60 s. In order to determine the chemical composition of the bonding zone, surface microanalyses were performed using an Oxford X-Max electro dispersive spectrometer (EDS) (Oxford Instruments, Abingdon, UK).

Additionally, experiments using Electron Backscatter Diffraction (EBSD) on a Scanning Electron Microscope (SEM) Hitachi Su70 (Hitachi High-Tech Corporation, Tokyo, Japan) have been carried out. EBSD measurements were taken at Warsaw University of Technology, Faculty of Materials Science and Engineering. Sample preparation was based on ion polishing on the Hitachi IM4000 device (Hitachi High-Tech Corporation, Tokyo, Japan). EBSD measurements were taken with an accelerating voltage of 20 kV and a step size of 100 nm. After measurements, the noise reduction step was carried out. Based on the measurements, the orientation maps (OIMs), phase, and grain boundary distribution maps have been analyzed. Low-angle grain boundaries (LAGB) were assumed to be boundaries with misorientation angles of at least 3° and up to 15°, while high-angle grain boundaries (HAGB) were boundaries above 15°.

### 2.3. Electrochemical Research

Potentiokinetic polarization curves were made in a 0.5 M Na_2_SO_4_ solution acidified to a pH = 4.0. Electrodes in the form of rotating discs were used for the tests, in which fragments of the side surfaces of the tested rods with an area of 0.2 cm^2^ were used as electrodes. All potentiodynamic tests were carried out at a temperature of 25 ± 0.1 °C, with the rotational speed of the disk equal to 12 rps-1 and the potential scanning speed 0.005 V∙s^−1^ using its shift from the value Einit 0.3 V lower than Ecor to Efin = +0.5 V (relative to AgCl/Ag). Each time, before plotting the potentiodynamic curve, the tested sample was kept in the corrosion solution for 15 min, i.e., until the corrosion potential reached a stationary value.

### 2.4. Microhardness Studies

The analysis was also supported by the Vickers microhardness distribution. The measurements were performed on a Struers DURA SCAN 70 microhardness tester (EMCO-TEST Prüfmaschinen GmbH, Kuchl, Austria) with a 0.98 N load. For each sample, a line of measurements has been taken, from the top surface to the distance of 9 mm.

## 3. Research Results

### 3.1. Microstructural Studies

**Figure 5:**
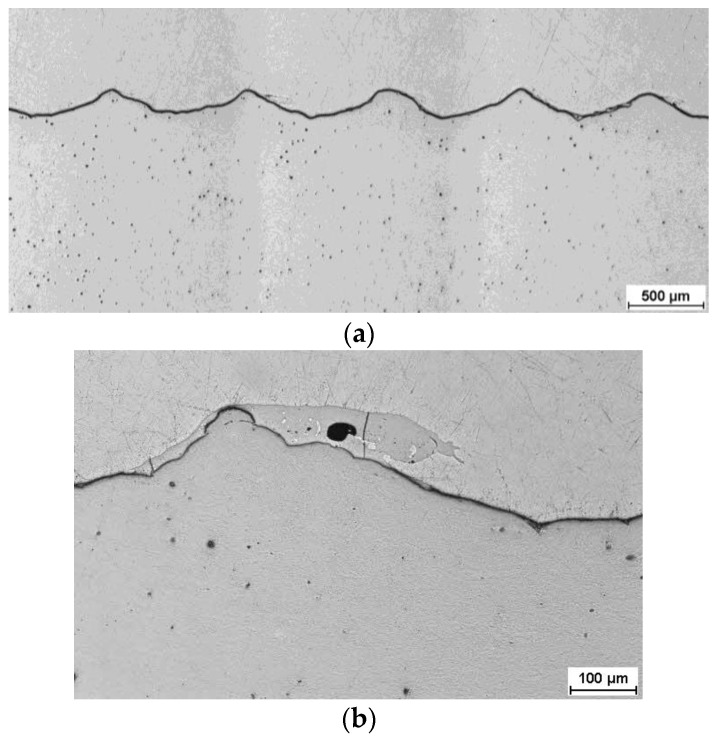
Microstructure of the joint area in a longitudinal section after explosive welding: (**a**) low magnification; (**b**) a single wave with a localized region of intermetallic phases (high magnification)—light microscope images.

To determine structural changes in individual components and the joint areas of Mg/Al rods after explosive welding and finished bimetallic rods following the rolling process in two variants, metallographic examinations were conducted. An example of the interface of the joint after explosive welding is shown in Figure 5.

**Figure 6:**
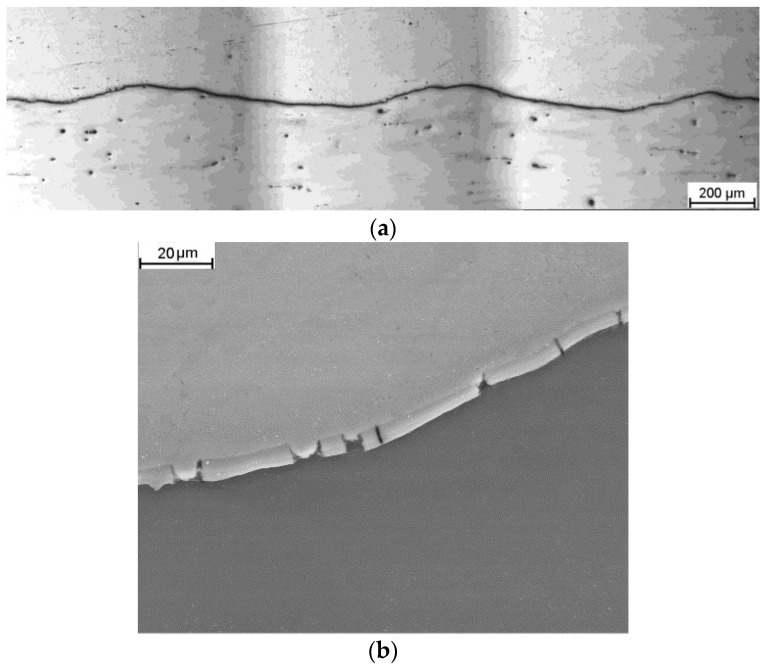
Microstructure of the joint area in a longitudinal section after rolling at a temperature of 300 °C: (**a**) low magnification; (**b**) a section of a single wave with an intermetallic phase layer (high magnification)—light microscope images.

**Figure 7:**
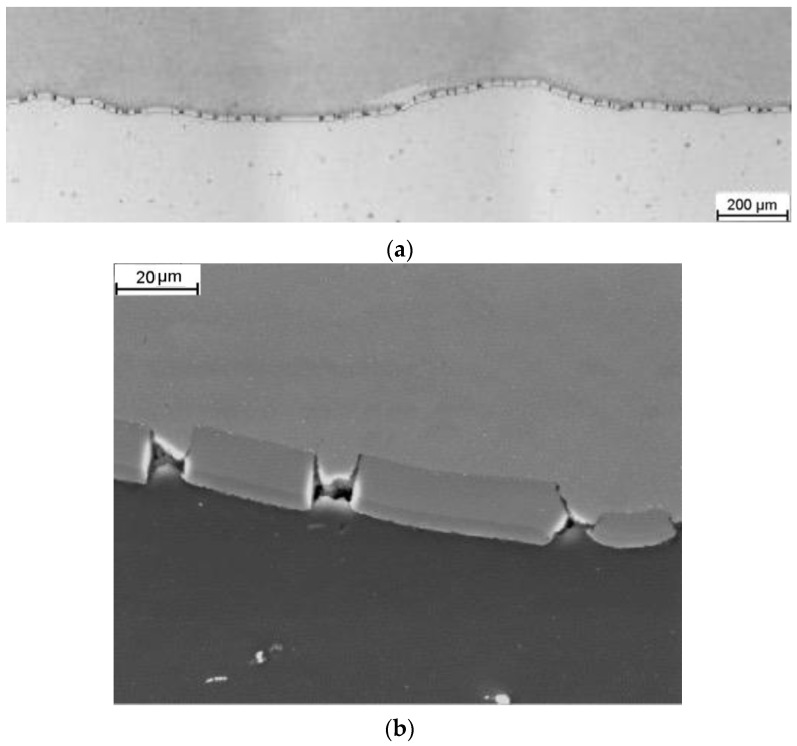
Microstructure of the joint area in a longitudinal section after rolling at a temperature of 400 °C: (**a**) low magnification; (**b**) a section of a single wave with an intermetallic phase layer (high magnification)—light microscope images.

In the longitudinal section at higher magnification, only localized intermetallic phases were observed at the interface. These phases do not form a continuous layer but rather localized areas of irregular shape. In none of the analyzed samples, the presence of a continuous zone of brittle Mg/Al intermetallic phases in the joint area was observed, which should result in a high coherency of the joint in bimetallic samples. Figure 6 and Figure 7 depict an exemplary microstructure of the joint areas for samples rolled in modified sections at various temperatures. After rolling at a temperature of 300 °C, a continuous, very thin (5–10 µm) layer of Mg/Al intermetallic phases is observed at the Mg/Al interface, as shown in Figure 6a. This layer formed during the rolling process. SEM analysis revealed cracks in this layer, as depicted in Figure 6b.

After rolling at a temperature of 400 °C, a continuous but significantly thicker (above 20 µm) layer of Mg/Al intermetallic phases is observed at the Mg/Al interface, as shown in Figure 7a. This layer also formed during the rolling process. SEM analysis also revealed cracks in this layer, as depicted in Figure 7b. For both analyzed samples, locally thicker, irregular areas of Mg/Al intermetallic phases were observed, which formed during the explosive welding process (Figure 7b). The waves underwent elongation and flattening due to sample elongation. The shape of the Al grooves did not significantly affect the joint area. The critical parameter was the temperature.

**Figure 8:**
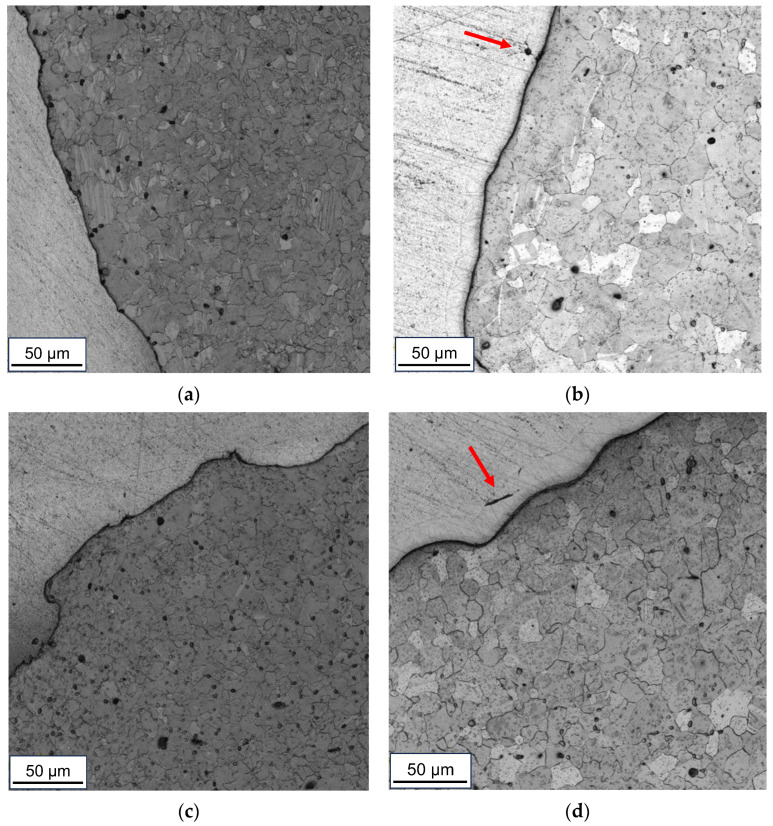
Microstructure of the AZ31 alloy of samples from: (**a**) Series I; (**b**) Series II; (**c**) Series III; (**d**) Series IV—light microscope images.

Analyzing the microstructure of the AZ31 alloy in the obtained samples, it can be concluded that the bimetals treated at a lower temperature (300 °C) are characterized by a finer grain. The bond zones of chemically etched samples are presented in Figure 8. In the case of the Series III sample, the grain size is approx. 20 μm. It is noteworthy that the sample obtained using the CMC pattern is characterized by both noticeable inhomogeneities in the grain size (there are both larger grains of 20 μm and finer ones with about 10 μm) and a strongly twinned microstructure. The share of twin grain boundaries decreases with the distance from the AZ31-AA1050 joint line. The samples deformed at a higher temperature (400 °C) are characterized by a slightly larger grain size with single, finer, healed grains. Also in this case, the CMC-formed sample (Series II) is characterized by a noticeable proportion of grain boundaries twinning, but to a lesser extent than the sample deformed at a lower temperature (Series I). In addition, in the samples from Series II and IV, the presence of a diffusion zone with local cracks (marked with red arrows) was found.

**Figure 9:**
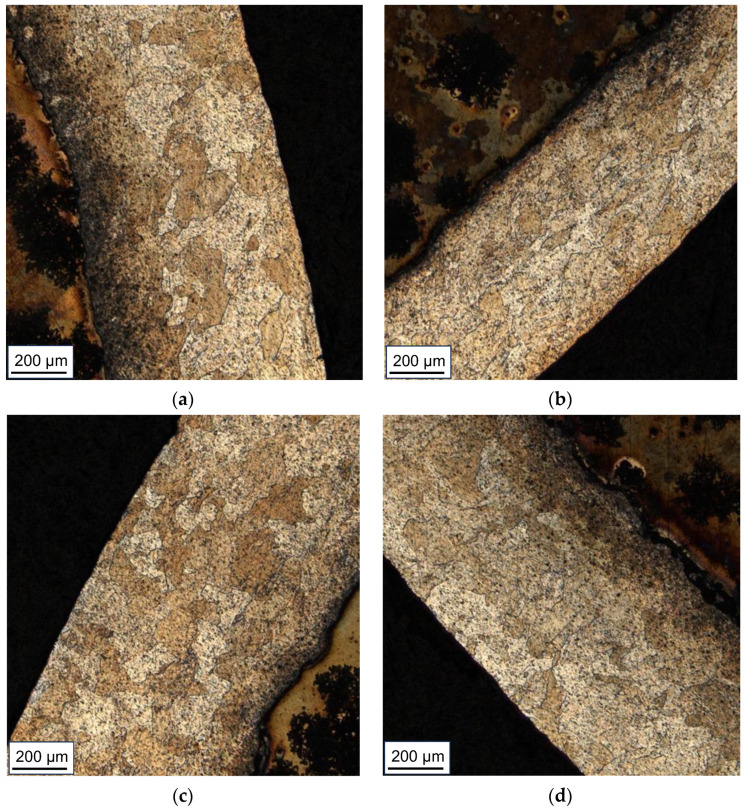
Microstructure of the AA1050 layer of samples: (**a**) Series I; (**b**) Series II; (**c**) Series III; (**d**) Series IV—light microscope images.

The microstructure of the AA1050 layers (Figure 9) is characterized by high heterogeneity and differences in the size of individual grains, being generally coarse-grained (100–200 μm). The samples from series I and III are characterized by a noticeable deformation of the aluminum layer located directly at the surface of the bimetallic rod, the most intense in the sample formed with the CMC pattern to a depth of approx. 100 μm. In the case of the sample from series IV, formed at 400 °C, no plastically deformed surface layer was found. The Series II sample is characterized by the presence of fine grains (approx. 10 μm) within the surface layer, suggesting the dynamic recrystallization process, as well as larger, healed grains.

**Figure 10:**
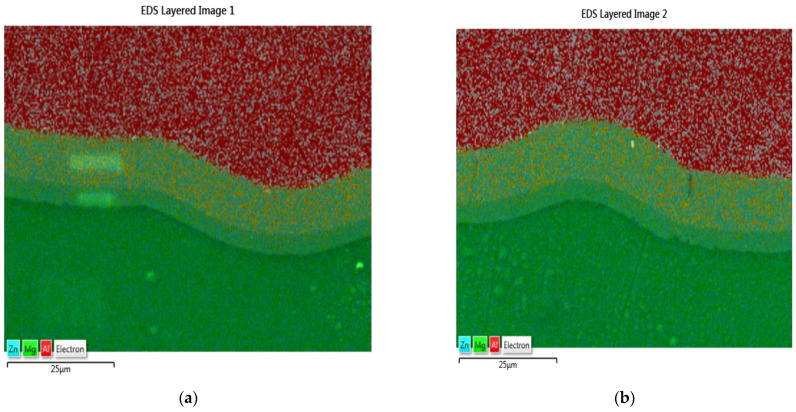
AZ31-AA1050 transition zone for samples: (**a**) Series II; (**b**) Series IV—SEM/EDS images.

Microstructural observations carried out using a scanning electron microscope (Figure 10) allowed for the identification of the diffusion zone in samples deformed at a temperature of 400 °C. Regardless of the type of sample, the diffusion zone is continuous and consists of two layers with different proportions of magnesium and aluminum. Earlier studies [31] conducted by the authors on deformation at elevated temperature of the AA1050-AZ31 junction indicate that the layers of the diffusion zone are composed of intermetallic phases, respectively: β (Mg_2_Al_3_) on the aluminum side and γ (Mg_17_Al_12_) on the magnesium side. In the case of the Series IV sample, the diffusion zone has a thickness of approx. 20 μm with the participation of individual phases of 14 μm Mg_2_Al_3_ and 6 μm Mg_17_Al_12_. In the Series II sample, the zone is slightly smaller and has a thickness of approx. 15 μm, with a share of 10 μm Mg_2_Al_3_ and 5 μm Mg_17_Al_12_. At the stage of microstructural studies, it was found that the single cracks were located mainly in the layers of the Mg_2_Al_3_ phases.

### 3.2. EBSD Studies

**Figure 11:**
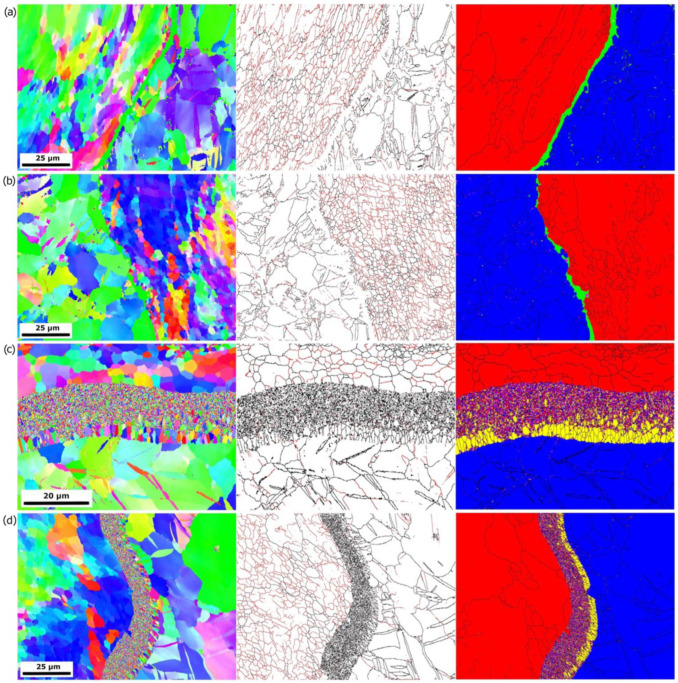
OIM together with a map of boundaries distribution (red line—LAGB; black line—HAGB) and HAGB + phases (red—Al; blue—Mg; yellow—Mg_17_Al_12_; pink—Al_3_Mg_2_; green—unsolved points) of the samples: (**a**) Series III; (**b**) Series I; (**c**) Series IV; (**d**) Series II—SEM/EBSD images.

EBSD results are shown in Figure 11. From OIM, the differences in grain size between the samples can be observed. The smallest grain size is seen for the samples from Series I and Series III, and it is growing for further samples. With increasing the rolling temperature, the grain size for both materials—Al and Mg—increases. For Al, a large number of LAGBs are observed. A smaller grain size is obtained for the Al compared to the Mg, where the majority of grain boundaries are HAGB. For all samples, regardless of the rolling temperature, the majority of grains for the Al are LAGB. However, for samples II and IV, the average size of grains/subgrains increases. In the case of Mg, coarser grains with observed twin boundaries are present in the microstructure. The most significant difference between the samples is observed at the interface between Al and Mg. For the Series I and Series III samples, this zone was not solved during the measurements (green areas in the phase map). Nevertheless, the thickness of this zone is not considerable and does not exceed 5 µm. For the Series II and Series IV samples, between Al and Mg, a thick layer has been observed. In the case of the Series IV sample, the maximum thickness of this zone is 17 µm, while for the Series II sample, the thickness varies from 10 to 20 µm. However, it must be noted that a more extensive region was examined in the case of the Series II sample. In this transition zone, two phases are distinguished—Al_3_Mg_2_ and Mg_17_Al_12_. The latter is placed next to the Mg phase, characterized by columnar, coarser grains with lengths up to 4 µm and 4.7 µm for the samples Series II and Series IV, respectively. As we approach the Al zone, the transition zone consists mainly of the Al_3_Mg_2_ phase, characterized by a refined, much smaller grain size. Nevertheless, the Al and Mg phases were also detected during the measurements in this area. The results are similar to what was observed in work [32], where three-layered Al/Mg/Al materials via explosive welding have been investigated. At the interfaces of the materials, two main precipitate types have been observed—Mg_17_Al_12_ and Mg_2_Al_3_. The Mg_17_Al_12_ precipitates had columnar morphology, while the Mg_2_Al_3_ precipitates were both columnar and equiaxial. Also, in work [33], where explosively welded AZ31B/AA6061 bimetals were investigated, the annealing process at a temperature above 250 °C resulted in the formation of intermetallic compounds of Mg_17_Al_12_ and Mg_2_Al_3_, which were formed at the bonding interface. As in the present study, Al_3_Mg_2_ was formed on the Al side, while Mg_17_Al_12_ was on the Mg side. Moreover, the thickness of intermetallic compounds was increasing with both—increasing the annealing temperature and time. Interesting findings were observed in [34], where it was shown that annealing leads to diffusion processes that homogenize the chemical composition of reaction regions, and various chemical compositions are systematically transformed into Mg_2_Al_3_. It could also explain the results in the present study and the unsolved areas shown in Figure 11a,b, as presumably some phases with different chemical compositions or a mixture of them could be observed (as in [34]) but not solved during EBSD measurements.

### 3.3. Microhardness Measurements

**Figure 12:**
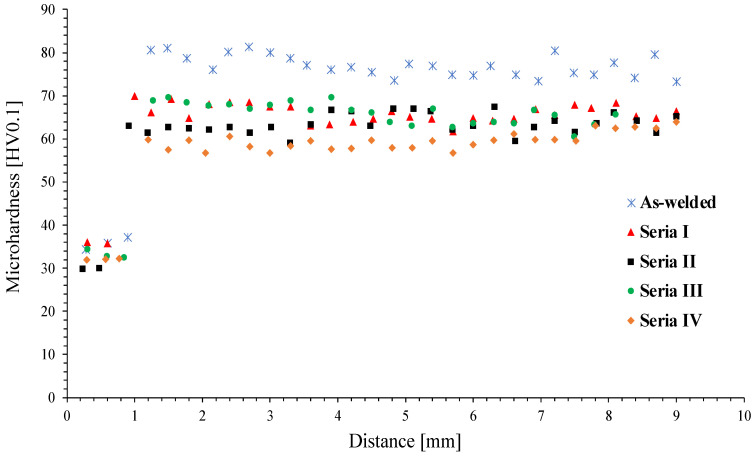
Microhardness distribution.

The observations made during the microstructural analysis are reflected in the obtained microhardness distributions. A microhardness distribution graph is presented in Figure 12. The highest values of microhardness, both layers AA1050 and AZ31, were obtained for bimetal samples treated at a lower temperature (300 °C): Series I and III, respectively, approx. 33 and 36 HV0.1. In the case of the Series I sample, characterized by a strong twinning of the microstructure of the AZ31 alloy within the joint line (Figure 8a), the recorded microhardness value is 70 HV0.1. The lowest strengthening values of the AA1050 layer were found for the sample from Series II with a microhardness of 30 HV0.1. The microhardness distributions in the AZ31 alloy between individual samples are characterized by noticeable discrepancies, and only at a depth of approx. 8 mm do they assume similar values. Similar to the AA1050 layer, the lowest microhardness values of the AZ31 alloy are found in samples formed at a higher temperature (400 °C), while the Series II sample is characterized by greater uniformity and microhardness. Overall, the application of higher temperatures during the deformation contributes to the decrease of material as-welded strengthening due to previously reported recrystallization processes (Figure 8c,d and Figure 11c,d). It has to be taken into consideration that greater deformation of grains makes heat-activated phenomena easy to occur, and for this reason, the observed changes predominantly concern the joint zone.

### 3.4. Electrochemical Research

**Figure 13:**
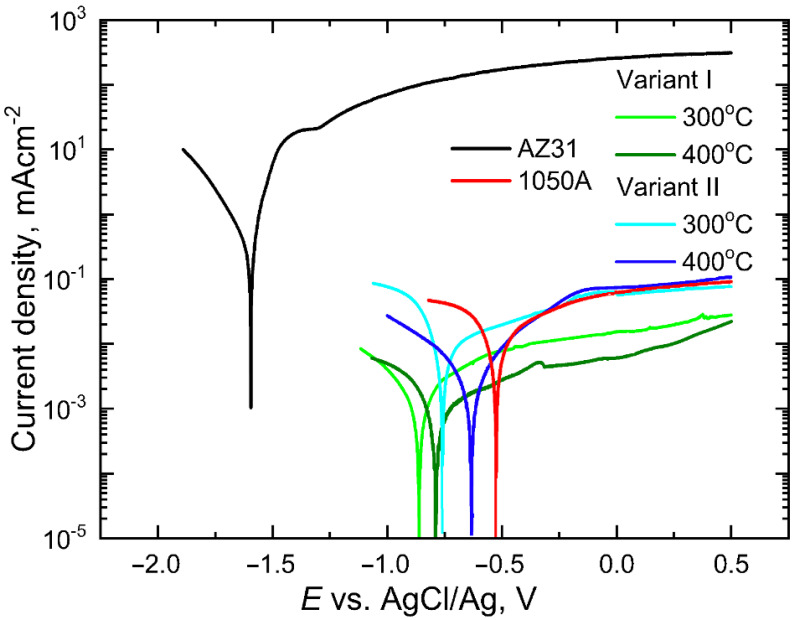
Potentiodynamic polarization curves for components in the initial state and Mg/Al bimetals rolled according to two variants at 300 °C or 400 °C, made in 0.5 M Na_2_SO_4_ with pH = 4; Experimental conditions: 25 ± 0.1 °C, potential scanning speed 0.005 Vs^−1^, disk rotation speed 12 rpm^−1^.

The obtained polarization curves plotted for materials in the initial state and for Mg/Al bimetals rolled according to two variants are shown in Figure 13. The experiment generated polarization curves for two sets of materials: AZ31 alloy bars in their original state and Mg/Al bimetals formed through two different rolling methods—the classic one (variant I) and the modified one (variant II). Despite the metals’ close positions in the voltage series (Mg at −2.37 V and Al at −1.66 V), the AZ31 alloy bars and 1050A aluminum tubes used as the base materials for making bimetals exhibited distinct corrosion characteristics when exposed to an acidic environment [35,36].

As it is known [35,37], the starting materials used to obtain the tested bimetals are characterized by different corrosion properties. Despite the short distance in the voltage series of metals (normal potential for Mg is −2.37 V and for Al: −1.66 V) in acidic environments, the magnesium-based alloy AZ31 undergoes intensive dissolution, while the aluminium alloy is covered with a passive layer, effectively protecting the surface of this material against corrosion [38]. These differences are clearly visible in Figure 13. In the applied corrosive environment, the magnesium alloy, which is the core of the bimetal, reaches anode currents more than three orders of magnitude higher than the aluminium alloy. For the AZ31 alloy, these currents (*i*_a_) reach values of approx. 300 mAcm^−^^2^, while for the aluminium alloy, they do not exceed 0.20 mAcm^−^^2^. Such low current values for aluminium in the anode range testify to strong inhibition of corrosion processes and effective protection of the ally surface by the resulting passive layer. Clear differences for the output components are also visible in the values of corrosion potentials (Figure 13). For the magnesium alloy, the corrosion potential (*E*_cor_) is low in the applied environment (*E*_cor_ = −1.6 V), which proves the rapid transition of this material to the state of active dissolution, while for the easily passivated aluminium it is definitely higher: −0.53 V. Comparing the courses of the bimetallic potentiodynamic curves presented in Figure 13, it can be seen that their courses are affected by both the temperature and the rolling method used (the shape of the grooves). For both rolling variants and for both temperatures, the shift of the corrosion potential (*E*_cor_) to values lower than the value of −0.53 V, characteristic for the 1050A alloy, is observed. Such a shift proves an earlier start of the dissolution of the surface of the aluminium alloy as a result of its deformation in the rolling process. It should be noted, however, that while the anode currents (*i*_a_) for rolling according to “variant II—modified rolling” do not change and remain at the level of 1050A alloy in the initial state, “variant I—classical rolling” of rolling ensured their reduction for both process temperatures used and thus facilitated the transition of the bimetal surface to the passive state. The exact values of *E*_cor_ and *i*_a_ read at *E* = 0.0 V are listed in Table 6. Table 6 also includes the values of the polarization resistance (*R*_p_) and the corrosion current density (*i*_cor_) calculated on its basis, which is a measure of the corrosion rate. According to the Stern-Hoar equation [39], for potentials slightly different from the corrosion potential, *E*_cor_ = ±20 mV, the external current density is a linear function of the potential, and the slope of the corresponding lines is a measure of the polarization resistance. In turn, the inverse of *R*_p_ is proportional to the corrosion current density. This dependence is valid for systems in which partial processes are controlled by activation, i.e., those for which the Tafel coefficients take strictly defined values (usually *b*_a_ = 0.03 ÷ 0.06 V and *b*_k_ = 0.12 V). Often, however, the Stern-Hoar relationship is also used in cases where the metal is passivated (*b*_a_ = ∞) or when the cathodic process is diffusion controlled (*b*_k_ = ∞). In our case, assuming formally that *b*_a_ = ∞ and *b*_k_ = 0.12 V, we find:

(1) $$i_{cor}=\frac{b_{a}b_{k}}{2.3(b_{a}+b_{k})}=\frac{0.052[V]}{R_{p}}$$

**Figure 14:**
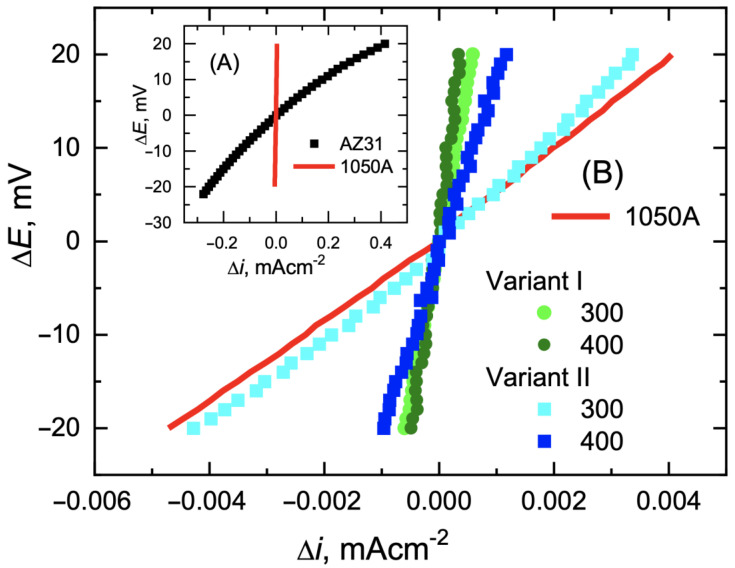
Measurements of linear polarization Δ*E* = *E* − *E*_cor_ = *f*(*iext*) of starting materials (**A**) and bimetals after rolling processes at 300 °C or 400 °C (**B**).

In Equation (1), the corrosion current density is expressed in [Acm^−^^2^] and the polarization resistance in [Ωcm^2^]. Figure 14 shows the relationship of linear polarization Δ*E* = *E* − *E*_cor_ = *f*(Δ*i*) for potentials equal to *E*_cor_ ± 20 mV for bimetals after various rolling variants.

As can be seen from the potentiodynamic diagrams (Figure 13) and the data collected in Table 6, bimetals after rolling processes show slightly more favourable corrosion parameters (lower values of *i*_cor_ and *i*_a_) than the aluminium alloy from which the cover was made. This slight improvement in corrosion parameters is probably due to the reduction in grain size of the material as a result of fragmentation of the primary grains during the work process. For the tested bimetals, for which the share of aluminium in the cross-section of the rod was 15%, the lowest corrosion rate (*i*_cor_ = 1.1 × 10^−^^3^ mA·cm^−^^2^) was recorded for variant I at a temperature of 400 °C. However, as the authors showed in their previous work [1], the corrosion processes of the tested bimetals can be slowed down even more by increasing the cover thickness in the cross-section of the bimetallic rod. For the share of 28% aluminium in the cross-section of the bimetal, the corrosion rate for modified rolling at 300 °C decreased to *i*_cor_ = 0.3 × 10^−^^3^ mA·cm^−^^2^.

## 4. Conclusions

On the basis of the obtained results, the following conclusions can be drawn:

1. This study determined the influence of process parameters on the pattern and the possibility of controlling the plastic flow of Mg/Al bimetallic rods during their rolling in elongating grooves.
2. Both rolling technologies ensure very good corrosion parameters for the Mg/Al bimetal. After the rolling process, the material shows clearly lower values of both *i*_cor_ and current in the passive range.
3. As a result of hot forming, the microhardness of AZ31 has been reduced (compared to the as-welded state) by 15 HV0.1 on average. The reduction in the AA1050 layer is significantly lower and reported only for the rods forming at 400 °C.
4. In the transition zone of bimetallic rods rolling at 400 °C, two phases are distinguished—Al_3_Mg_2_ and Mg_17_Al_12_, localized next to the Mg core, characterized by columnar, coarser grains with lengths within the range of 4–5 µm. Closer to the Al layer, the transition zone consists mainly of the Al_3_Mg_2_ phase, characterized by refined, smaller grain sizes. With a 15 share of the cover layer in the cross-section of the bimetallic rod, “variant I—classical rolling” of rolling slows down the dissolution rate slightly more and facilitates the transition of the surface of this material to a passive state.

## Figures and Tables

**Table 1:** Chemical composition of the materials used for the tests.

Material	Chemical Composition, % Mass.								
AZ31	Mn	Mg	Cu	Zn	Ca	Al	Si	Fe	Ni
	0.24	rest	–	0.72	–	2.8	0.01	0.003	0.001
AA1050	Si	Fe	Cu	Mn	Mg	Zn	Ti	Al	Pb
	0.06	0.18	0.002	0.003	0.002	0.008	0.020	99.74	–

**Table 2:** The dimensions of the tubes and bars used for the explosive welding process.

No. of Set/Sample	Outer Diameter of Al Tube [mm]	Inner Diameter of Al Tube [mm]	Wall Thickness [mm]	Diameter of the Mg Bar [mm]	Distance between Al Tube and Mg Bar [mm]
1, 2, 3	24	21	1.5	19.2	0.9
4 ÷ 10	23	21	1.0	19.2	0.9

**Table 3:** Average dimensions of Mg-Al bimetallic bars after explosive welding.

Average External Diameter [mm]	Average Proportion of Al Layer in the Cross-Section [%]	Average Thickness of Al Layer [mm]
21.2	16.8	0.93 ± 0.02

**Table 4:** Types and designations of tested materials.

Type of Material	Sample Designation
Aluminium alloy AA1050	100% Al
Magnesium alloy AZ31	AZ31
Bimetals form after explosive welding	−AZ31 + 15%Al
Bimetals after classical rolling, respectively at 300 °C or 400 °C—Variant I	Series I—rolling temperature: 300 °C Series II—rolling temperature: 400 °C
Bimetals after modified rolling, respectively at 300 °C or 400 °C—Variant II	Series III—rolling temperature: 300 °C Series IV—rolling temperature: 400 °C

**Table 5:** Thickness distribution of the plating layer obtained in Mg/Al bars after rolling.

Temp.	Constituting	Bimetallic Rods		Variant I—Classical Rolling		Variant II—Modified Rolling		Changes
[°C]	[%]	Average thickness of the layer [mm]	K_plat_	Average thickness of the layer [mm]	K_plat_	Average thickness of the layer [mm]	K_plat_	[%]
300	15	0.93	1.351	0.78	1.444	0.79	1.36	6.4
400	15	0.93	1.351	0.73	1.574	0.76	1.51	4.1

**Table 6:** The most important parameters determining the corrosion resistance of starting materials and Mg/Al bimetals after various rolling variants.

Material/Rolling	Rolling Temperature	*E*_cor_, [V]	*i*_a_ [mA·cm^−2^]	*R*_p_ [Ω·cm^2^]	*i*_cor_ [mA·cm^−2^]
AZ31		−1.6	260	60	860 × 10^−3^
Variant I	300 °C	−0.86	1.5 × 10^−2^	30 × 10^3^	1.7 × 10^−3^
	400 °C	−0.78	0.6 × 10^−2^	50 × 10^3^	1.1 × 10^−3^
Variant II	300 °C	−0.75	6.0 × 10^−2^	5.8 × 10^3^	8.9 × 10^−3^
	400 °C	−0.63	6.5 × 10^−2^	20 × 10^3^	2.6 × 10^−3^
1050 A		−0.53	6.0 × 10^−2^	5.5 × 10^3^	9.5 × 10^−3^

## Data Availability

Not applicable.
